# Different responses of colorectal cancer cells to alternative sequences of cetuximab and oxaliplatin

**DOI:** 10.1038/s41598-018-34938-y

**Published:** 2018-11-08

**Authors:** Elli Narvi, Katri Vaparanta, Anna Karrila, Deepankar Chakroborty, Sakari Knuutila, Arto Pulliainen, Maria Sundvall, Klaus Elenius

**Affiliations:** 1 Institute of Biomedicine and Medicity Research Laboratories, University of Turku, Turku, Finland; 2 Turku Doctoral Programme of Molecular Medicine, Turku, Finland; 3 Department of Pathology, Haartman Institute, University of Helsinki, Helsinki, Finland; 4 Department of Oncology, Turku University Hospital, Turku, Finland

## Abstract

Therapeutic protocols including EGFR antibodies in the context of oxaliplatin-based regimens have variable clinical effect in colorectal cancer. Here, we tested the effect of the EGFR antibody cetuximab in different sequential combinations with oxaliplatin on the growth of colorectal cancer cells *in vitro* and *in vivo*. Cetuximab reduced the efficacy of oxaliplatin when administered before oxaliplatin but provided additive effect when administered after oxaliplatin regardless of the *KRAS* or *BRAF* mutation status of the cells. Systemic gene expression and protein phosphorylation screens revealed alternatively activated pathways regulating apoptosis, cell cycle and DNA damage response. Functional assays indicated that cetuximab-induced arrest of the cells into the G1 phase of the cell cycle was associated with reduced responsiveness of the cells to subsequent treatment with oxaliplatin. In contrast, oxaliplatin-enhanced responsiveness to subsequent treatment with cetuximab was associated with increased apoptosis, inhibition of STAT3 activity and increased EGFR down-regulation. This preclinical study indicates that optimizing the sequence of administration may enhance the antitumor effect of combination therapy with EGFR antibodies and oxaliplatin.

## Introduction

Cancer cells accumulate genetic alterations leading to atypical regulation of a wide variety of signaling networks including those regulating apoptosis, cell cycle, growth factor receptor signaling, and DNA damage response. The interconnected network of cancer cell signaling routes can be readjusted using drugs activating or inhibiting these networks leading to adaptive cellular responses. The optimal design of combination therapy is dictated by the genetic background of the cells and needs understanding of how the complex networks are reorganized following treatments with single compounds or combinations of drugs^1,2^.

Monoclonal antibodies (mAb) targeting the epidermal growth factor receptor (EGFR), cetuximab and panitumumab, have been approved for the treatment of *RAS* wild-type metastatic colorectal cancer (CRC). Both drugs have demonstrated clinical benefit as single agents, as well as in combination with irinotecan- or oxaliplatin-based chemotherapies^3^, while the efficacy of cetuximab in different regimens containing oxaliplatin and non-infusional fluoropyrimidine has also been questioned^4,5^.

When combined with oxaliplatin, the EGFR mAbs are routinely administered on day 1 of the clinical treatment cycle, before oxaliplatin infusion. However, the optimal sequencing of the EGFR mAb/oxaliplatin combination remains to be determined. Some preclinical studies have suggested that the administration of EGFR inhibiting compounds after cytotoxic agents increases efficacy^6–9^, while others have indicated that pretreatment with an EGFR inhibitor sensitizes cells to DNA-damaging drugs^1,10^. Given the strong impact of genetic background on the optimal sequencing of drugs for breast cancer cells^1^, it is also possible that CRC cells with alternative mutation status respond differently to alternative sequential treatments.

Here, we assessed the efficacy of EGFR mAbs in simultaneous and sequential combinations with oxaliplatin in a panel of colorectal cancer cell lines with different genetic backgrounds (wild-type or mutant for *KRAS* or *BRAF*). The administration of cetuximab after oxaliplatin treatment was more efficient when compared to administration of cetuximab before oxaliplatin in all cell lines tested. Molecular studies addressing the adaptive mechanisms readjusting signaling routes during the alternate sequential regimens revealed significant differences in the pathways regulating apoptosis, cell cycle, and DNA damage response.

## Results

### Sensitivity of colorectal cancer cell lines to cetuximab and oxaliplatin

**Figure 1:**
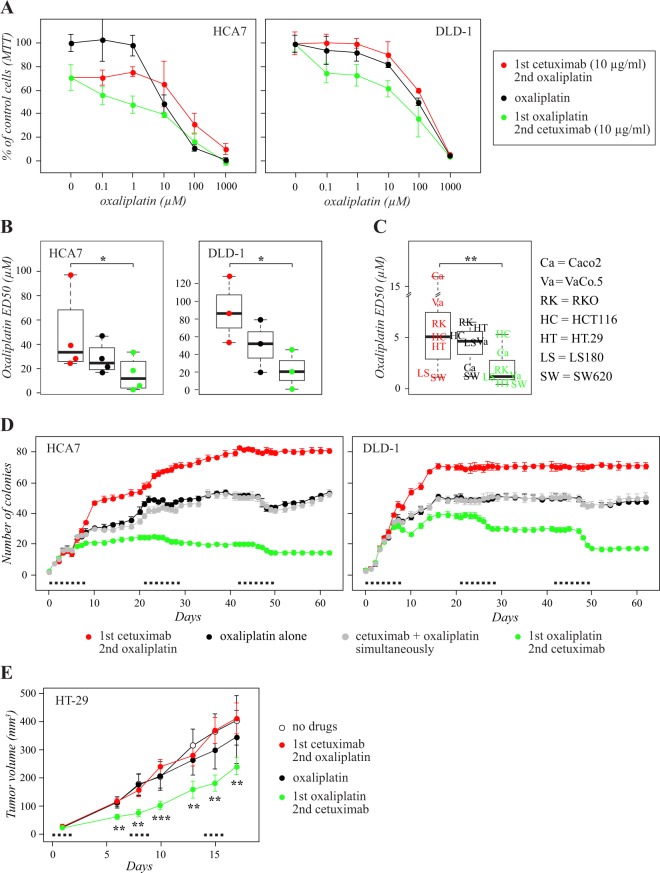
Sequence of administration determines the effect of the regimen including cetuximab and oxaliplatin. Sequential administration of cetuximab before or after oxaliplatin was compared to administration of oxaliplatin alone using MTT (**A**–**C**), soft agar (**D**), and mouse xenograft (**E**) growth assays. (**A**–**C)** Cells were (1) treated for 24 hours (for day 3 of the experiment) with the indicated concentrations of oxaliplatin alone (black curves), (2) treated first for 48 hours (days 1 and 2) with 10 μg/ml cetuximab followed by 24 hour (day 3) treatment with the indicated concentrations of oxaliplatin (red curves), or (3) treated first for 24 hours (day 3) with oxaliplatin followed by 48 hour (days 4 and 5) treatment with 10 μg/ml cetuximab (green curves). MTT analysis was carried out after day 5 of the experiment. (**A**) Representative MTT experiments of HCA7 (left panel) and DLD-1 (right panel) cells. Mean +/− SD is shown. (**B**) Oxaliplatin ED50 values of sequential treatments from 3–4 independent experiments with HCA7 and DLD-1 cells. (**C**) Oxaliplatin ED50 values of sequential treatments from single independent experiments with the seven indicated cell lines. The box plots indicate the median, the second and third quartiles, and the range of the data. *P < 0.05; **P < 0.01. (**D)** HCA7 (left panel) and DLD-1 (right panel) cells suspended in soft agar were (1) treated for 48 hours (for days 4 to 5 of each treatment cycle) with oxaliplatin alone (black curves), or (2) in simultaneous combination with cetuximab (grey curves), (3) treated first for 72 hours (days 1 to 3) with cetuximab followed by 48 hour (days 4 to 5) treatment with oxaliplatin (red curves), or (4) treated first for 48 hours (days 4 to 5) with oxaliplatin followed by 72 hour (days 6 to 8) treatment with cetuximab (green curves). The cycle was repeated three times every 21 days as indicated by black dots. The concentrations used for oxaliplatin and cetuximab were 50 µM and 10 µg/ml, respectively. Mean +/− SD is shown. (**E**) Nude mice carrying HT-29 cell xenografts were treated with i.p. injections of (1) vehicle alone on day 1 of each treatment cycle (white dots), (2) oxaliplatin alone on day 1 (black dots), (3) cetuximab on day 1 followed by oxaliplatin on day 2 (red dots), or (4) oxaliplatin on day 1 followed by cetuximab on day 2 (green dots). The cycle was repeated three times every seven days as indicated by black dots. The concentrations used for oxaliplatin and cetuximab were 10 mg/kg and 40 mg/kg, respectively. Mean +/− SEM is shown. **P < 0.01; ***P < 0.001, for a comparison between the two alternative sequences (green *vs*. red curves).

To address the effect of EGFR mAbs and oxaliplatin on the growth of CRC cells, nine ATCC-derived cell lines (HCA7, Caco-2, LS180, SW620, DLD-1, HCT116, HT-29, RKO, and VaCo-5) were characterized for their *KRAS*, *NRAS*, and *BRAF* mutation status and tested for sensitivity to single agent cetuximab, panitumumab or oxaliplatin using MTT cell viability assay (Table 1; Suppl. Fig. 1A). All cell lines were wild-type for *NRAS*, consistent with previous findings^11^ (www.broadinstitute.org/ccle/home). Four of the cell lines (HCA7, LS180, HCT116 and RKO) carry wild-type p53 protein while the others harbor mutations in the *TP53* gene (www.p53.free.fr). Of the two cell lines wild-type for both *KRAS* and *BRAF*, HCA7 was growth-inhibited by both tested EGFR mAbs at ED50 < 0.1 µg/ml (Table 1; Suppl. Fig. 1A). The other one, Caco-2, was resistant to EGFR mAbs as defined by the ED50 exceeding 100 μg/ml (Table 1). In contrast, with the exception of one cell line, LS180, harboring a *KRAS* Gly12Asp mutation as well as a *BRAF* Asp211Gly mutation, all the *KRAS* or *BRAF* mutant lines were resistant to 100 μg/ml of both EGFR mAbs (Table 1; Suppl. Fig. 1A). All the nine cell lines responded to single agent oxaliplatin with ED50 values ranging from 1.2 to 72 µM (Fig. 1B,C).

**Table 1:** KRAS and BRAF mutation status and ED50 values for cetuximab (μg/ml) of the studied CRC cell lines.

cell line	*KRAS*	*BRAF*	ED50 (µg/ml) cetuximab
HCA7	wt	wt	0.02 ± 0.1
Caco-2	wt	wt	>100
LS180	Gly12Asp	Asp211Gly	0.004 ± 0.002
SW620	Gly12Val	wt	>100
DLD-1	Gly13Asp	wt	>100
HCT116	Gly13Asp	wt	>100
HT-29	wt	Val600Glu	>100
RKO	wt	Val600Glu	>100
VaCo-5	wt	Val600Glu	>100

Wt, wild-type. Mean +/− SD is shown.

The possible additive effect of combining EGFR mAbs with oxaliplatin was analyzed by adding cetuximab to cells simultaneously with increasing concentrations of oxaliplatin, and comparing cell viability to treatment with oxaliplatin alone. However, while the cetuximab-sensitive cell lines demonstrated some increased responsiveness, no significant additive effect was observed when the analysis was carried out for all the nine cell lines with variable *KRAS*/*BRAF* mutation status (Suppl. Fig. 1B and data not shown).

### Sequential administration of cetuximab and oxaliplatin

To address whether sequential drug administration differed from simultaneous combination, HCA7 (*KRAS* wild-type, *BRAF* wild-type) and DLD-1 (*KRAS* mutant, *BRAF* wild-type) cell lines were subjected to three different treatment regimens: (1) oxaliplatin alone, (2) first treatment with cetuximab followed by oxaliplatin, or (3) first treatment with oxaliplatin followed by cetuximab. The sequential regimen “cetuximab after oxaliplatin” was significantly more effective than the opposite regimen “cetuximab before oxaliplatin” in both HCA7 and DLD-1 cells (*P* = 0.021 and 0.015, respectively) (Fig. 1A,B). As the finding indicated that the efficacy of “cetuximab after oxaliplatin” was not restricted to the *KRAS*/*BRAF* wild-type background, the experiment was repeated using a panel of seven other colorectal cancer cell lines, representing variable *KRAS*/*BRAF* genotypes (Table 1). Consistent with the findings of HCA7 and DLD-1 cells, the sequential regimen “cetuximab after oxaliplatin” was more effective than the opposite regimen “cetuximab before oxaliplatin” also in an analysis of seven additional cell lines (P = 0.0015) (Fig. 1C). A similar sequential regimen test was reproduced by replacing oxaliplatin with irinotecan. However, no significant differences were observed between different sequences of EGFR mAb and irinotecan administration in HCA7 or DLD-1 lines (Suppl. Fig. 2).

In the clinical practice, the drugs are given in repeated cycles. To simulate the cyclic scheduling, the activity of the sequential administration was analyzed in *in vitro* tumor formation assays with HCA7 and DLD-1 cells growing in soft agar. The cells were subjected to different oxaliplatin- and cetuximab-containing sequential or simultaneous regimens that were repeated every 21 days for three cycles. As in the MTT cell viability assays, simultaneous addition of cetuximab to oxaliplatin did not result in significantly increased activity (*P* = 1.0 for HCA7, *P* = 0.37 for DLD-1), but the two sequential regimens were significantly different when compared to each other, oxaliplatin alone, or to simultaneous cetuximab plus oxaliplatin (*P* < 0.001 for all comparisons for both HCA7 and DLD-1) (Fig. 1D). During the three 21 day cycles of the experiment, the cells seemed to become less and less sensitive to the other three regimens while no *in vitro* resistance developed for the sequence of cetuximab after oxaliplatin (Fig. 1D).

### Effects of sequences on xenograft tumor growth *in vivo*

To address the effects of the sequential treatment protocols on *in vivo* tumor growth, HT-29 cells were grown as xenografts in immunocompromised female nude mice. The HT-29 cell line was chosen as a model based on its relatively efficient growth as mouse xenograft. The genetic profile of HT-29 cells represents the *KRAS* and *NRAS* wild-type tumor subtype, but includes the activating V600E mutation in the *BRAF* gene (Table 1). The mice were inoculated with 3 × 10^6^ HT-29 cells by subcutaneous injections followed by three weekly cycles of intraperitoneal administration of drugs. For each one week cycle, the mice were given injections of buffer alone, oxaliplatin alone, cetuximab followed by oxaliplatin, or oxaliplatin followed by cetuximab. Similar to the *in vitro* findings, the administration of cetuximab after oxaliplatin was significantly more efficient that cetuximab administered before oxaliplatin in suppressing xenograft growth (Fig. 1E).

### Regulation of cell cycle and apoptosis

**Figure 2:**
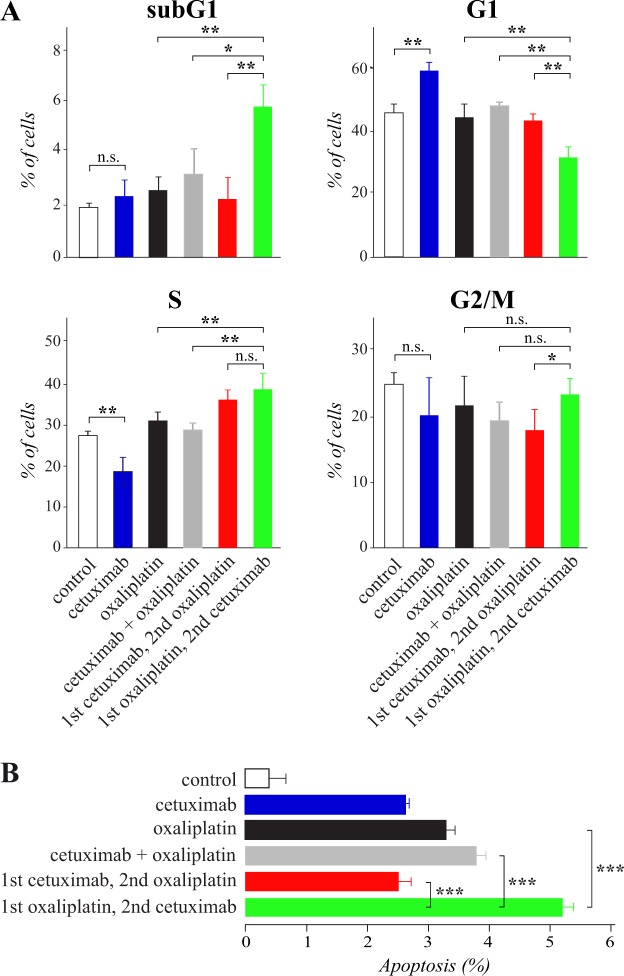
Sequential administration of cetuximab after oxaliplatin reduces G1 arrest and enhances apoptosis. Cell cycle (**A**) and apoptosis (**B**) analyses of HCA7 cells subjected to different regimens containing 10 μg/ml cetuximab and/or 50 μM oxaliplatin: (1) control medium for 24 hours (white), (2) cetuximab for 24 hours (blue), (3) oxaliplatin for 24 hours (black), (4) simultaneous combination of oxaliplatin and cetuximab for 24 hours (grey), or treated sequentially (5) first with cetuximab for 24 hours followed by oxaliplatin for 24 hours (red) or (6) first with oxaliplatin for 24 hours followed by cetuximab for 24 hours (green). Cell cycle was analyzed by PI staining (**A**) and apoptosis by annexin V staining (**B**). Mean +/− SD is shown.

To address the cellular mechanisms underlying the differential effects of the two sequences, the *RAS* wild-type HCA7 cells were treated with different regimens containing cetuximab or oxaliplatin as single agents or as sequential or simultaneous combinations as indicated in Fig. 2. After the treatments, the cells were subjected to cell cycle and apoptosis analyses by PI and annexin V staining, respectively.

The cell cycle analyses indicated that cetuximab treatment alone accumulated cells into the G1 phase but cetuximab administered after oxaliplatin was unable to induce G1 arrest (Fig. 2A). Instead, “cetuximab after oxaliplatin” appeared to efficiently accumulate cells into the S and subG1 phases. Given the increase of cells in the subG1 fraction, it is also possible that some of the cells categorized into the S phase in response to “cetuximab after oxaliplatin” actually represented cells with less than duplicated DNA amount as they were entering apoptosis while arrested in G2/M^12^. Nevertheless, treatment with the opposite sequence “cetuximab before oxaliplatin” resulted in significantly fewer cells in the G2/M phase as compared to “cetuximab after oxaliplatin” (Fig. 2A).

Consistent with the differential accumulation of cells into the subG1 fraction in the cell cycle analysis (Fig. 2A), “cetuximab after oxaliplatin” was also significantly more efficient in promoting apoptosis than “cetuximab before oxaliplatin” when the percentage of early apoptotic cells was analyzed by annexin V staining (Fig. 2B). “Cetuximab after oxaliplatin” was significantly more potent in promoting apoptosis also when compared to “oxaliplatin alone” based on both the PI and annexin V staining analyses (Fig. 2A,B).

### Differential gene expression patterns

**Figure 3:**
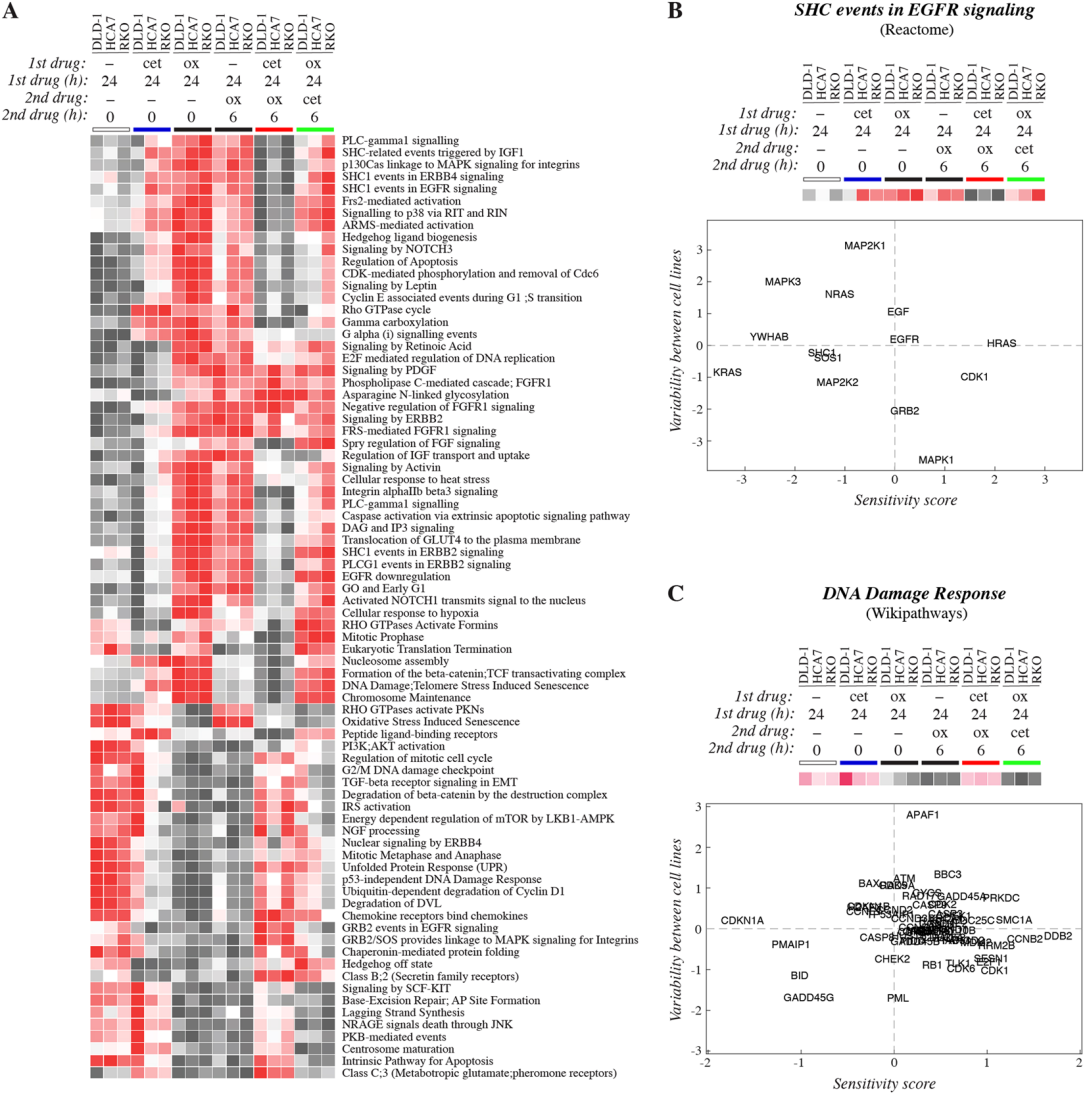
RNAseq analysis of transcriptomic profiles induced by sequential treatments. DLD-1, HCA7, and RKO cells were subjected to different regimens containing 10 μg/ml cetuximab and/or 50 μM oxaliplatin: (1) control medium for 24 hours (white bar), (2) cetuximab for 24 hours (blue bar), (3) oxaliplatin for 24 hours (left black bar), (4) control medium for 24 hours followed by oxaliplatin for 6 hours (right black bar), (5) cetuximab for 24 hours followed by oxaliplatin for 6 hours (red bar), or (6) oxaliplatin for 24 hours followed by cetuximab for 6 hours (green bar). Gene expression was analyzed using RNAseq. (**A**) Data were sectioned into pathway matrices according to the annotations of the Reactome database and converted into a heatmap. (**B**,**C**) Sensitivity scores reflecting changes in the expression of single genes in response to the different treatment protocols were plotted against the variation in the expression between the three different cell lines. Data are shown for sets of genes constituting two pathways, one reflecting differential responses to EGFR manipulation (“SHC events in EGFR signaling”) (**B**), and the other differential responses to the DNA damage induction by oxaliplatin (“DNA Damage Response”) (**C**). The two pathways were based on annotations by the Reactome (**B**) and Wikipathways (**C**) databases also shown as heatmaps in panel A and Suppl. Fig. 5, respectively.

To characterize the dynamic adaptations in molecular signaling routes during sequential drug treatments at the gene expression level, an RNAseq analysis was performed. Since the differential effect of the sequences was independent on the *KRAS*/*BRAF* status of the cells (Fig. 1A–C), the experiment was carried out in three cellular backgrounds representing the different genotypes: HCA7 (*KRAS* wild-type, *BRAF* wild-type), RKO (*KRAS* wild-type, *BRAF* mutant), and DLD-1 (*KRAS* mutant, *BRAF* wild-type). The cells were treated with six different regimens as indicated in Fig. 3A. Gene expression data were sectioned into pathways using annotations by Reactome, Wikipathways and Biocarta databases, and pathway scores were determined to find significantly differentially regulated signaling cascades between the treatments. The data obtained using the Reactome database is shown in Fig. 3A, as well as with full annotation codes in Suppl. Fig. 3, and the data obtained with the Wikipathways and Biocarta databases in Suppl. Figs 4 and 5, respectively.

Treatment of the HCA7 and RKO cells with cetuximab alone promoted clear differences in the pathway-specific expression signature when compared to control cells treated with medium alone. Interestingly, the only *KRAS* mutant cell line, DLD-1, responded to cetuximab by an expression pattern more reminiscent to that of the control cells, consistent with the role of activating *RAS* mutations as conferring resistance to EGFR mAbs^13,14^. Treatment with oxaliplatin alone (for 6 or 24 hours) resulted in even more widespread change in the expression landscape than treatment with cetuximab alone, when compared to the control without treatment. As expected based on the functional assays indicating reduced effect of oxaliplatin by prior exposure to cetuximab, the treatment with “cetuximab before oxaliplatin” largely prevented the changes induced by oxaliplatin alone. In contrast “cetuximab after oxaliplatin” mostly recapitulated or modulated the changes promoted by oxaliplatin alone (Fig. 3A; Suppl. Figs 3–5). Specific pathways that were differentially regulated between the regimens included those mediating apoptosis, DNA damage response, or cell cycle checkpoint pathways, as well as those involved in relaying signals of specific molecular cascades, such as EGFR, MAPK, TGF-beta receptor, or Wnt signaling pathways (Fig. 3A; Suppl. Figs 3–5).

### Regulation of single genes

The pathways visualized by the heatmap presentations were based on expression of selected genes with presumed functional roles on the pathway. To illustrate how the heatmaps reflect the responses of the cells to the different regimens, sensitivity scores indicating variation in the expression of individual genes (collectively constituting the pathway) in response to all the six treatment protocols were determined. The sensitivity scores were then plotted against a score reflecting the variability in the expression between the three cell lines tested. As an example of such analysis, data from a pathway reflecting responses to targeting EGFR with cetuximab (“SHC events in EGFR signaling”), as well as from a pathway reflecting the response to the DNA damage inducing oxaliplatin (“DNA Damage Response”) are shown (Fig. 3B,C).

Both sensitivity score analyses included genes that were clearly differentially expressed between the protocols and thus predominantly contributed to the changes observed in the heatmap analyses. For example, differentially expressed genes in the “DNA Damage Response” pathway included those encoding the cell cycle regulator p21 (*CDKN1A*), as well as its targets CDK2, CDK4 and CDK6, cyclins B2 (*CCNB2*) and D1 (*CCND1*), and the DNA damage-associated factors DNA damage-binding protein 2 (*DDB2*), GADD45 gamma (*GADD45G*), Structural maintenance of chromosomes protein 1A (*SMC1A*), and DNA-dependent protein kinase catalytic subunit (*PRKDC*) (Fig. 3C). The expressional changes of apoptosis regulators NOXA (*PMAIP1*) and BH3 interacting-domain death agonist (*BID*) also affected the “DNA Damage Response” pathway score (Fig. 3C).

To validate the RNAseq pathway analysis, a trained model based on measured apoptosis of the HCA7 cells, as well as on the two Reactome pathways “Intrinsic Pathway for Apoptosis” and “Caspase Activation via Extrinsic Apoptotic Signaling Pathway”, was created to predict apoptosis in the two other cell lines included in the RNAseq analysis, DLD-1 and RKO. A set of ten selected genes derived from the two pathways were indeed identified that predicted the same differential apoptotic responses to the treatments as observed for the HCA7 cells (Suppl. Fig. 6). As a control, the actual apoptosis of DLD-1 and RKO cells in response to the different treatment regimens was also measured by annexin V staining. As expected, “cetuximab after oxaliplatin” promoted significantly more apoptosis when compared to “cetuximab before oxaliplatin” in both DLD-1 and RKO cells (Suppl. Fig. 6D).

Taken together, these examples demonstrate that the cetuximab- and oxaliplatin-containing regimens provoked transcriptional responses in pathways relevant for the regulation of cell cycle, DNA damage, and apoptosis.

### Regulation of signaling proteins

**Figure 4:**
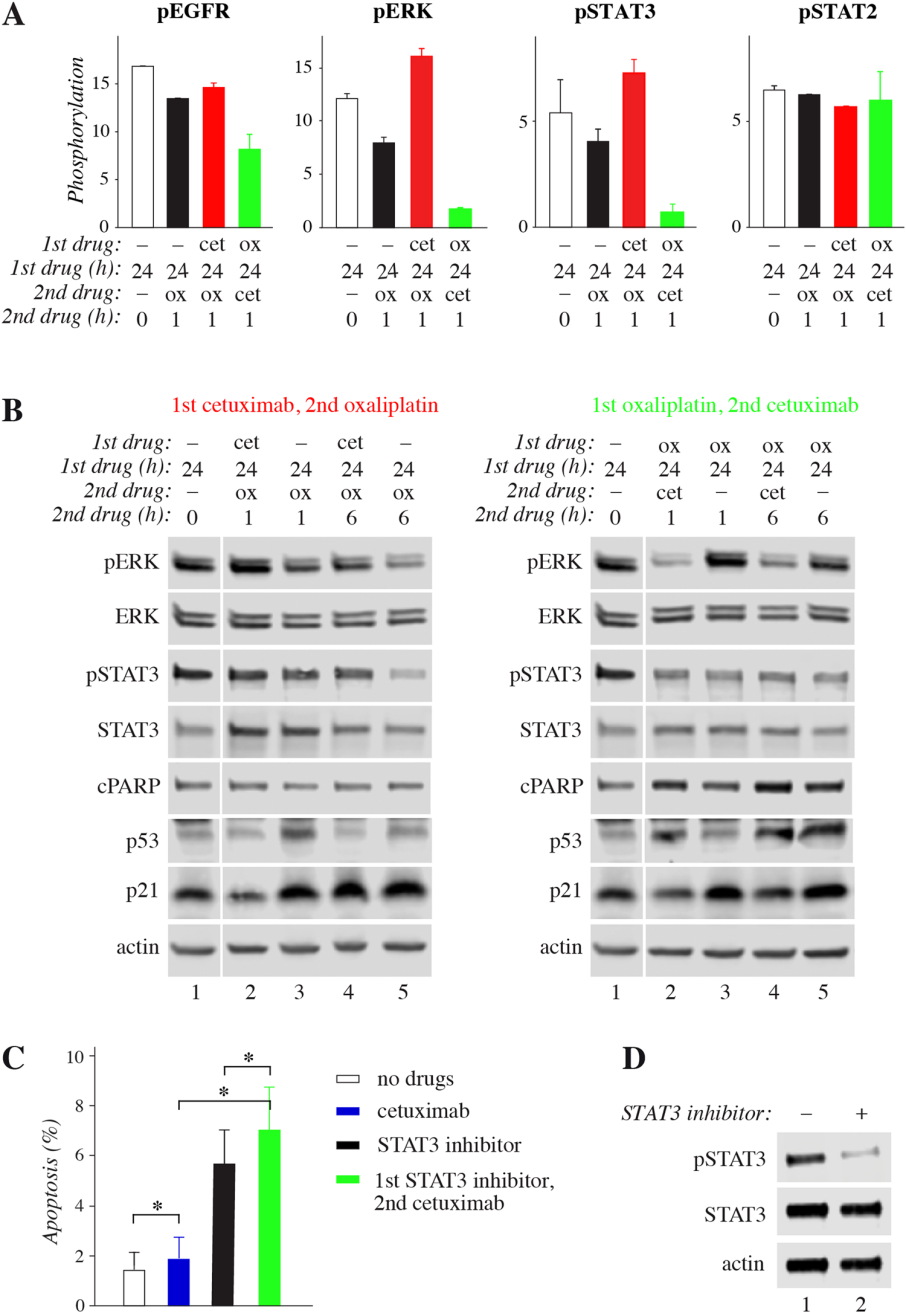
Phosphoarray analysis of signaling responses to sequential treatments. (**A**) HCA7 cells were subjected to different regimens containing 10 μg/ml cetuximab and/or 50 μM oxaliplatin: (1) control medium for 24 hours (white), (2) control medium for 24 hours followed by oxaliplatin for 1 hour (black), (3) cetuximab for 24 hours followed by oxaliplatin for 1 hour (red), or (4) oxaliplatin for 24 hours followed by cetuximab for 1 hour (green). Phosphorylation profiles were analyzed using Proteome Profiler Human Phospho-Kinase Array Kit and quantified by densitometry. Mean +/− range is shown for four selected proteins. All results are shown in Suppl. Fig. 3. (**B**) HCA7 cells were subjected to different regimens containing or not 10 μg/ml cetuximab and/or 50 μM oxaliplatin as indicated in the figure. The expression or phosphorylation of selected proteins was analyzed by Western blotting. Full-length blots are presented in Supplementary Fig. 8. (**C**) Apoptosis of HCA7 cells was analyzed by annexin V staining after treating the cells first with or without 200 μM of the STAT3 inhibitor S31-201 for 8 hours followed by 18 hour treatment with or without 10 μg/ml cetuximab. Mean +/− SD is shown for three independent experiments. *P < 0.05.

To address the activation of cellular signaling cascades following the sequential drug treatments, phosphorylation of 45 different kinases and other signaling molecules was analyzed using the Proteome Profiler Human Phospho-Kinase Array Kit. To this end, HCA7 cells were treated with oxaliplatin alone, or the two sequential protocols including oxaliplatin and cetuximab as indicated in Fig. 4A.

When compared to the control cells treated with medium alone or to the opposite sequence, the administration of “cetuximab after oxaliplatin” demonstrated clearly reduced phosphorylation of EGFR, as well as of some of its major downstream signaling molecules including ERK, AKT, STAT3, and SRC family kinases (Fig. 4A; Suppl. Fig. 7). In contrast, no significant changes were observed in the phosphorylation of some other signaling molecules, such as STAT2 or the heat-shock protein HSP27 (Fig. 4A; Suppl. Fig. 7). The opposite sequence “cetuximab before oxaliplatin”, in turn, reduced the phosphorylation of GSK3 but induced the phosphorylation of CREB when compared to the opposite sequence and control cells (Suppl. Fig. 7).

To corroborate the experimentation carried out with the RNAseq and phosphoarray screens, expression and phosphorylation of selected signaling molecules was further analyzed by Western analysis in time points shown in Fig. 4B. Consistent with the phosphoarray data, inhibition of EGFR activity by cetuximab clearly reduced ERK phosphorylation regardless of oxaliplatin pretreatment (Fig. 4B, right panel) while “cetuximab before oxaliplatin” increased the pERK levels when compared oxaliplatin alone (Fig. 4B, left panel). As expected based on the apoptosis and cell cycle analyses (Fig. 2), “cetuximab after oxaliplatin” increased PARP cleavage, a molecular readout of cell death^15^, when compared to oxaliplatin treatment alone (Fig. 4B, right panel). The steady-state level of the p53 protein that senses cellular stress^16^ was also clearly up-regulated by the cytotoxic oxaliplatin, while cetuximab administered before oxaliplatin reduced p53 accumulation (Fig. 4B, left panel). No similar protective effect was observed when cetuximab was administered after oxaliplatin (Fig. 4B, right panel). Consistent with the contribution of the p21-encoding *CDKN1A* gene on the “DNA Damage Response” pathway in the RNAseq heatmap analysis (Fig. 3C), p21 protein level also reacted to the different sequences. p21 protein was accumulated in response to oxaliplatin alone, but the response was rapidly reversed by addition of cetuximab (Fig. 4B, right panel), putatively also adding to the apoptotic response of cells to “cetuximab after oxaliplatin”.

In accordance with the phosphoarray data (Fig. 4A), as well as previous observations^17^, the level of phosphorylated STAT3 was clearly reduced by long (>1 hour) oxaliplatin treatment, and this reduction was not affected by subsequent administration of cetuximab (Fig. 4B). However, cetuximab added before oxaliplatin significantly attenuated the effect (Fig. 4B, left panel, lane 4 *vs*. 5). Interestingly, the STAT3 pathway has previously been reported to be critical for colorectal cancer cell growth promoted *via* EGFR^18^. To functionally address whether suppression of STAT3 activity could replace oxaliplatin in inducing an additive effect with cetuximab, a chemical STAT3 inhibitor, S31-201, was used. Indeed, treatment of HCA7 cells with the STAT3 inhibitor significantly potentiated the apoptotic effect of subsequently added cetuximab (Fig. 4C), suggesting that inhibition of STAT3 phosphorylation by oxaliplatin could be involved as one mechanism leading to more activity when the EGFR mAb cetuximab is administered after oxaliplatin. Taken together, these findings indicate that the differential cellular responses promoted by the two EGFR mAb/oxaliplatin sequences are regulated by molecular signaling pathways regulating apoptosis and cell cycle.

### Mechanistic model

**Figure 5:**
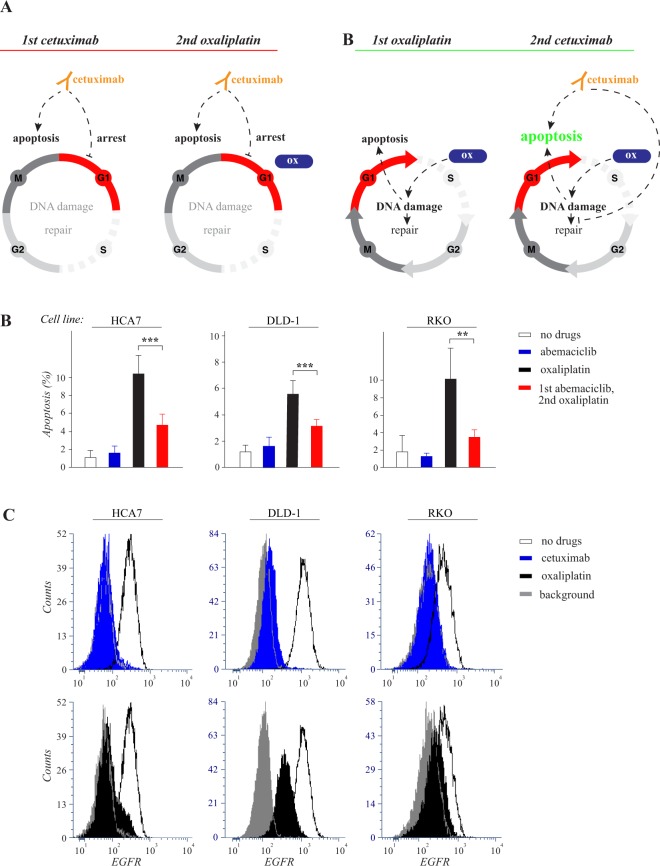
Oxaliplatin is less efficient in G1-arrested cells but induces EGFR downregulation. (**A**) Schematic representation of hypothetical cellular responses to the opposite sequences of cetuximab and oxaliplatin. *Left side*: When cetuximab is administered first, it promotes arrest in the G1 phase of the cell cycle and stimulates apoptosis by blocking EGFR-activated survival pathways. The subsequent administration of oxaliplatin (ox) is largely ineffective due to the G1-arrest. *Right side*: When oxaliplatin is administered first, it actively induces DNA damage provoking apoptosis, as well as DNA repair that partially compensates for the damage. In the absence of cetuximab, the cell cycle is not arrested at G1 allowing for full activity of oxaliplatin. However, oxaliplatin-induced DNA damage itself does eventually cause cell cycle arrest by activating checkpoints in the S and G2 phases (not indicated in the figure). When cetuximab is then added after oxaliplatin, apoptosis is enhanced compared to all other regimens, as the apoptotic effect of both drugs are active, and also, as cetuximab may be suppressing the DNA repair mechanisms caused by oxaliplatin-induced DNA damage. (**B**) HCA7, DLD-1 and RKO cells were arrested in G1 by 8 hour treatment with or without 3 µM of the CDK4/6 inhibitor abemaciclib followed by 18 hour treatment with or without 50 μM oxaliplatin. Apoptosis was measured by annexin V analysis. Mean +/− SD is shown for three independent experiments. **P < 0.01; ***P < 0.001. (**C**) Flow cytometry analysis of cell surface EGFR after 24 hour treatment with or without 10 μg/ml cetuximab or 50 μM oxaliplatin.

As cetuximab arrested HCA7 cells into the G1 phase of the cell cycle (Fig. 2A) and the DNA damage response has been shown vary between the different phases^19,20^, a hypothetical model can be generated in which “cetuximab before oxaliplatin” leads to an attenuated effect as it induces cell cycle arrest in G1 giving the cell time to repair the damaged DNA (Fig. 5A, left). To experimentally address this hypothesis – using the three cell lines modelling wild-type and *KRAS* and *BRAF* mutant colorectal cancer – the cells were treated with abemaciclib, a clinically approved CDK4/6 inhibitor, also known to arrest cell cycle in the G1/G0 phases^21^. Similar to cetuximab, abemaciclib administered before oxaliplatin resulted in significantly reduced apoptosis (Fig. 5B). Moreover, also cell cycle arrest induced at the G1/S border by treatment with thymidine^22^ suppressed HCA7 cell apoptosis induced by subsequent treatment with oxaliplatin (Suppl. Fig. 9). These findings, consistent with the observed regulation of CDK4 and CDK6 in the RNAseq screen (Fig. 3C), indicate that drugs, such as EGFR mAbs, that regulate cell cycle progression, may reduce the activity of subsequently administered oxaliplatin (Fig. 5A left).

While the model explains the negative effect of “cetuximab before oxaliplatin” it also accommodates with a paradigm for the positive effect of “cetuximab after oxaliplatin”, as in this sequence oxaliplatin is active prior to any cell cycle arrest caused by cetuximab. In this more effective sequence the two drugs would be free to act additively or synergistically in the absence of reciprocal interference (Fig. 5A, right). Examples of such additive effects were shown to result in enhanced arrest in G2/M (Fig. 2A) and apoptosis (Fig. 2B; Suppl. Fig. 6C) *via* combinatory activities on pathways involving cell cycle checkpoint, DNA repair, and apoptotic regulators (Figs 3 and 4; Suppl. Fig. 6). To further experimentally validate one possible level of positive interaction of the two drugs, EGFR down-regulation was addressed. “EGFR downregulation” was identified as one of the hits in the Reactome database analyses of the relevant transcriptionally regulated pathways (Fig. 3A). To this end, HCA7, DLD-1 and RKO cells were treated for 24 hours with cetuximab or oxaliplatin and down-regulation of endogenous cell surface EGFR was analyzed by FACS. Indeed, both cetuximab and oxaliplatin significantly reduced cell surface EGFR (Fig. 5C) providing an example of a converging molecular mechanism leading to reduced cell growth downstream of both drugs.

## Discussion

Here, we analyzed the effect of drug sequencing on the response of CRC cell lines to a treatment with an EGFR mAb and oxaliplatin. Our data indicated that, regardless of the *KRAS* or *BRAF* mutation status of the cells, the schedule in which the EGFR mAb cetuximab was given after oxaliplatin was superior to the sequence of cetuximab administered before oxaliplatin. Our mechanistic studies with functional assays measuring apoptosis, cell cycle progression, as well as phosphoarray and transcriptomic analyses highlighted the significance of differential regulation of apoptotic, cell cycle regulating, and DNA repair pathways in determining the response to the different sequential protocols.

Observations based on the phosphoarray and RNAseq analyses indicated that “cetuximab before oxaliplatin” was relatively ineffective maintaining similar signaling cascades as control treatments without drugs. These findings were consistent with the functional assays that did not demonstrate significant cytotoxicity of the sequence that was mostly inferior to single agent oxaliplatin. The cell cycle analyses with HCA7 cells indicated that the mechanism by which administration of cetuximab before oxaliplatin reduced the potency of the regimen involved cetuximab-promoted arrest of cells into the G1 phase of the cell cycle, where oxaliplatin was shown to be less effective (Fig. 5B). The cell cycle analysis also indicated that cetuximab treatment before oxaliplatin relatively reduced the progression of the cells into the G2/M phase, possibly as a result of the preceding cetuximab-induced arrest in the G1 phase.

The regimen “cetuximab after oxaliplatin” in turn was associated by increased apoptosis when compared to either the opposite sequence or single agent oxaliplatin, as demonstrated by annexin V staining, PARP cleavage analysis, and RNAseq expression-based analysis of apoptotic pathways. As our phosphoarray and RNAseq analyses of signaling cascades did not indicate interference of cetuximab with molecular events induced by prior oxaliplatin, it is possible that the two drugs, when given in the sequence “cetuximab after oxaliplatin”, function largely independently resulting in an additive effect (Fig. 5A).

EGFR blockade has been shown to induce apoptosis, independently of the mechanisms promoting cell death after DNA damage, for example by suppressing signaling *via* survival pathways, such as the PI3K/AKT pathway^23^. In addition to additive mechanisms, cetuximab given after oxaliplatin may also more directly modulate responses to the DNA-damaging oxaliplatin. Inhibition of EGFR signaling after inducing DNA damage by oxaliplatin may for example enhance apoptosis by suppressing the DNA repair mechanisms or survival pathways necessary for survival upon oxaliplatin-induced DNA-damage^24,25^ (Fig. 5B). Oxaliplatin was also shown to reduce EGFR from the cell surface, putatively as part of the cells adaptation to DNA damage response when cell division is not preferred. The remaining EGFR signaling may be needed for cell survival and the following EGFR blockage by cetuximab thus lead to apoptosis.

One candidate molecule for relaying molecular interactions between the pathways responding to EGFR inhibition and oxaliplatin is p21, a known negative regulator of both the cell cycle and apoptosis^26^. DNA damage induces the accumulation of p21 protein leading to arrest of the cell cycle at the G1/S or G2/M checkpoints in order to give cell time for DNA repair in both p53 dependent and independent mechanisms^26^. In our analyses, oxaliplatin arrested the cell cycle to the S phase and correspondingly induced p21 expression. Addition of cetuximab after oxaliplatin downregulated p21 levels rapidly, consistent with increased apoptosis when the cells continue DNA replication or enter mitosis without DNA damage repair.

Our findings indicated that the sequence “cetuximab after oxaliplatin” was effective also in cell lines harboring activating *KRAS* or *BRAF* mutations suggesting the involvement of pathways other that the RAS/RAF/MAPK cascade. p21 also binds to and represses the transcription factor STAT3^27^ and oxaliplatin was observed to clearly reduce STAT3 phosphorylation. Consistent with the hypothesis that modulation of the STAT3 pathway by oxaliplatin sensitizes the cells to EGFR inhibition^18,28,29^, chemical inactivation of the STAT3 pathway was shown to enhance cetuximab-induced apoptosis. These findings about the positive influence of oxaliplatin treatment on the responsiveness of cells to cetuximab provide a further possible layer of molecular interplay that may contribute to the superior apoptotic response to “cetuximab after oxaliplatin”. Furthermore, they could also partly explain the efficacy of the sequence in *KRAS* mutant cell lines resistant to single agent cetuximab.

Cancer therapy with targeted compounds is largely based on combinations with cytotoxic drugs. Continuing research is needed to optimize the timing and dosing of the drug combinations, as well as to identify biomarkers that predict the sensitivity not only to single agent drugs but also to different combination therapies. To our knowledge, this is the first preclinical analysis of the responsiveness of CRC lines with determined *RAS* and *BRAF* mutation status to sequential EGFR mAb/oxaliplatin combinations. While the exact molecular mechanisms underlying our observations remain to be elucidated, these findings suggest that the administration of cetuximab after oxaliplatin should be studied in the clinical setting as an option to treat metastatic CRC. It is of note, however, that the *in vivo* xenograft experiment was carried out with a colorectal cancer line, HT-29, that is wild-type for *KRAS* and *NRAS*, but harbors the activating V600E mutation in the *BRAF* gene. Although consistent with our *in vitro* observations about the independence of the results of the *RAS*/*BRAF* mutation status of the cell lines, clinical implications of the findings should be interpreted with caution, as cetuximab is typically avoided in the clinical practice for the treatment of the *BRAF* mutant cancer with relatively poor prognosis^3^. Moreover, careful optimization of the dosing schedule for the clinical setting will also be critical and needs to take into consideration the relatively long half-life of cetuximab that could affect oxaliplatin responses regardless of the cycle in which cetuximab is administered.

## Materials and Methods

### Cell culture

ATCC-derived human colorectal cancer cell lines HCA7, Caco-2, LS180, SW620, DLD-1, HCT116, HT-29, RKO, and VaCo-5, were maintained in DMEM supplemented with 10% FBS.

### Drugs and inhibitors

Oxaliplatin was purchased from Hospira Ltd, irinotecan from Teva, cetuximab (Erbitux) from Merck, panitumumab (Vectibix) from Amgen, abemaciclib (LY2835219) from Abmole, STAT3 inhibitor (S3I-201) from Cayman chemicals, and thymidine from Sigma-Aldrich.

### Mutation analysis

*KRAS*, *NRAS* and *BRAF* mutation status of the cell lines was determined using the Broad Institute’s Cancer Cell Line Encyclopedia (www.broadinstitute.org/ccle/home). *KRAS* (NCBI Gene ID: 3845) and *BRAF* (NCBI Gene ID: 673) hotspot mutation status was confirmed using DxS TheraScreen KRAS PCR Kit (Qiagen) and AmoyDx BRAF V600E Mutation Detection Kit (Amoy Diagnostics), respectively.

### MTT cell viability assay

Cells were plated at 5000 cells/well on 96-well-plates in triplicates and incubated for 24 hours before addition of drugs. After the treatments, the amount of viable cells was measured using the Promega CellTiter 96 Non-radioactive Cell Proliferation Assay. When tested as single agents or in simultaneous combinations, the cells were incubated for 72 hours in the presence or absence of 0.01–100 μg/ml cetuximab or panitumumab and/or 0.1–1000 μM oxaliplatin or 0.1–1000 μM irinotecan. When tested for sequential administration, the cells were incubated for 48 hours in the presence of 10 μg/ml cetuximab. After a wash with PBS, the cells were incubated for 24 hours in the presence of 0–1000 μM oxaliplatin. After another wash with PBS, cells that did not receive cetuximab prior to oxaliplatin were incubated for 48 hours in the presence of 10 μg/ml cetuximab, while cells that had received cetuximab were incubated for 48 hours. The oxaliplatin treatment was fixed to the day 3 of the experiment to administer the toxic drug to cells at a similar density in all three regimens.

### Soft agar growth assay

HCA7 and DLD-1 cells were plated at 120,000 cells/well on 6-well plates in triplicates in soft agar (0.6% agar for lower gel, 0.44% agar for upper gel with cells). Colonies, defined as at least eight cells in one cluster, were counted under the microscope every other day. The drugs were first administered after the appearance of the first colonies (day 1 of the experiment). A treatment cycle consisted of treatment with 50 μM oxaliplatin for 48 hours either alone or in simultaneous combination with 10 μg/ml cetuximab, or cetuximab administered for 72 hours before or after oxaliplatin treatment for 48 hours. The cells were washed by a one hour incubation in fresh medium each time the treatment was changed and after the last treatment of the cycle. The treatment cycle was repeated three times, starting on days 1, 21 and 42.

### Xenograft tumor model

Three million HT-29 cells in 100 µl of PBS were inoculated into the right flank of 8 weeks old female athymic Foxn1^nu^ nude mice (Envigo). Tumors were allowed to grow until palpable (for 4 days) before first drug injections. Mice were treated weekly with or without oxaliplatin (10 mg/kg i.p.), or sequential combination of oxaliplatin (10 mg/kg i.p.) and cetuximab (40 mg/kg i.p.) with cetuximab given either 24 hours before or 24 hours after oxaliplatin. Tumor volume was calculated using the formula 0.5*length*width^2^. Ten mice were analyzed for each treatment group. All animal experiments were approved by the Regional State Administrative of Southwestern Finland (license number: ESAVI/1694/04.10.07/2015), and carried out according to the regulations of the Finnish Act on Animal Experimentation (62/2006). The experiment was discontinued when first tumors reached the maximum diameter defined in the license (12 mm).

### Propidium iodide (PI) staining for cell cycle analysis

HCA7 cells were plated at 200,000 cells/well on 6-well plates. The following day, the cells were subjected to treatment protocols as indicated in Fig. 2A. The cells were harvested by trypsinization, washed twice with PBS and suspended to PI solution (0.05 mg/ml PI, 0.3% Triton X-100, 40 mM sodium citrate in PBS). Samples were analyzed by FACS.

### Apoptosis assay

Cells were plated at 250,000 cells/well on 6-well plates. The following day, the cells were subjected to treatment protocols as indicated in Figs 2, 4 and 5. Cells were trypsinized, washed once with PBS and once with annexin binding buffer (10 mM HEPES, pH 7.4, 140 mM NaCl, and 2.5 mM CaCl_2_). The cell pellets were suspended in 100 µl annexin binding buffer containing five μl of annexin V Alexa Flour 488 conjugate (Life Technologies) and PI solution at the final concentration of 10 μg/ml, followed by incubation for 15 minutes in the dark. 400 μl of annexin binding buffer was added before the samples were analyzed by FACS.

### Analysis of cell surface EGFR

Cells were plated at 250,000 cells/well on 6-well plates. The following day, the cells were subjected to treatment protocols as indicated in Fig. 5C. Cells were trypsinized, washed with PBS, incubated with anti-EGFR (sc-120; Santa Cruz Biotechnology) for 20 min on ice and with PE-conjugated anti-mouse antibody (Abcam) for 15 min on ice and analyzed by FACS.

### Phosphorylation screen

HCA7 cells were plated at 2.6 × 10^6^ cells/10 cm dish. The following day, cells were treated or not with 50 µM oxaliplatin or 10 µg/ml cetuximab for 24 hours, followed by one hour treatments with the alternative drug to test different sequences as indicated in Fig. 4A and Suppl. Fig. 7. After treatments, the phosphorylation status of cell lysates was analyzed using Proteome Profiler Human Phospho-Kinase Array Kit (R&D Systems ARY003B), as recommended by the manufacturer.

### Western blotting

HCA7 cells were plated at 2 × 10^6^ cells/10 cm dish in DMEM + 1.5% FBS. The following day, the cells were subjected to different treatment protocols, as indicated in Fig. 4B. Cells were lysed in lysis buffer (10 mm Tris-HCl, pH 7.4, 1% Triton-X100, 150 mm NaCl, 1 mm EDTA, 10 mm NaF) supplemented with 2 mm PMSF, 10 mg/ml aprotinin, 10 mg/ml leupeptin, 10 mM Na_4_P_2_O_7_ and 1 mm Na_3_VO_4_), and analyzed by Western blotting with the following primary antibodies: anti-ERK (#9102), anti-pERK (#9101), anti-PARP (#9542), anti-pSTAT3 (#9145), anti-STAT3 (#9139), anti-p21 (#2947) (all from Cell Signalling Technologies), anti-p53 (sc-126), and anti-actin (sc-1616) (both from Santa Cruz Biotechnology). The proteins were visually detected by infrared imaging using Odyssey CLx (LI-COR, Nebraska, US).

### RNAseq screen

HCA7, RKO, and DLD-1 cells were plated at 2.5 × 10^5^, 3 × 10^5^, or 1.7 × 10^5^ cells/well, respectively, on 6-well plates. The following day, the cells were subjected to different treatment protocols as indicated in Fig. 3 and Suppl. Figs 3–5. Cells were washed with PBS between all drug treatments. RNA was isolated using Nucleospin RNA kit (Macherey-Nagel) and sequenced by HiSeq 2500 equipment using single-end sequencing chemistry with 50 bp read length in one lane. The pathway analysis was conducted following the previously described underlying principles^30,31^. The derived gene counts were TPM pseudolog-transformed and sectioned into pathway matrices according to the selected pathway annotations of Reactome (reactome.org), Wikipathways (wikipathways.org) and Biocarta (biocarta.com) databases. Matlab implementation (https://lvdmaaten.github.io/drtoolbox/) of MCML (Maximally Collapsing Metric Learning)^32^ was used to derive the dimensionality reduced pathway score for each sample, which were pairwise tested using T-statistics to find the significantly differentially regulated pathways between treatment comparisons. The FDR-corrected *P*-value threshold was 0.001 for Reactome, 0.0005 for Wikipathways, and 0.001 for Biocarta pathway annotations. The sensitivity score for a specific gene in the pathway was calculated by multiplying the MCML weight coefficient for the gene with the mean of the gene TPM value to account for the influence in the pathway score. The projections of the first dimension were used to represent the variability between treatments and the second to ascribe the variability arising from diversity between cell lines.

### Statistical analyses

The ED50 values for drug effects were calculated from three parameter log logistic curves fitted to the corresponding MTT values. The MTT values were normalized with a non-parametric empirical Bayes method^33^ to exclude cell line variability and batch effects. Differences between sequential treatments were analyzed by comparing the slopes of the fitted log logistic curves with a z-test. The acquired *P*-values were FDR-corrected for multiple testing error. In the flow cytometry analyses, soft agar and xenograft experiments, student’s t-test was used. *P* < 0.05 was accepted as statistically significant.

## Electronic supplementary material

Dataset 1
