# Supplementary material for: Different responses of colorectal cancer cells to alternative sequences of cetuximab and oxaliplatin
Source: Sci Rep. 2018 Nov 8;8:16579. doi: 10.1038/s41598-018-34938-y (PMC6224565; doi:10.1038/s41598-018-34938-y)
Supplement: Supplementary file 1 — Dataset 1 [file 41598_2018_34938_MOESM1_ESM.pdf]

Different responses of colorectal cancer cells to alternative sequences of cetuximab and oxaliplatin

Elli Narvi[1], Katri Vaparanta[1,2], Anna Karrila[1,2], Deepankar Chakroborty[1,2], Sakari Knuutila[3], Arto Pulliainen[1], Maria Sundvall[1,4], and Klaus Elenius*[1,4]

[1]Institute of Biomedicine and Medicity Research Laboratories, University of Turku, Finland
[2]Turku Doctoral Programme of Molecular Medicine, Turku, Finland
[3]Department of Pathology, Haartman Institute, University of Helsinki, Finland
[4]Department of Oncology, Turku University Hospital, Turku, Finland

Supplementary materials and methods

Apoptosis prediction model. To create the apoptosis prediction model, the HCA7-derived RNAseq data from genes from the Reactome pathways R-HSA-109606 and R-HSA-5357769 and the experimental annexin V apoptosis data were used as a training set for a polynomial support vector machine. The trained model was used to predict the amount of apoptosis in the RKO and DLD-1 cell lines from the RNAseq data. The sensitivity of each gene in the model was determined by setting the mean gene expression value for each treatment sequentially for each gene one at a time and running it through the prediction algorithm.

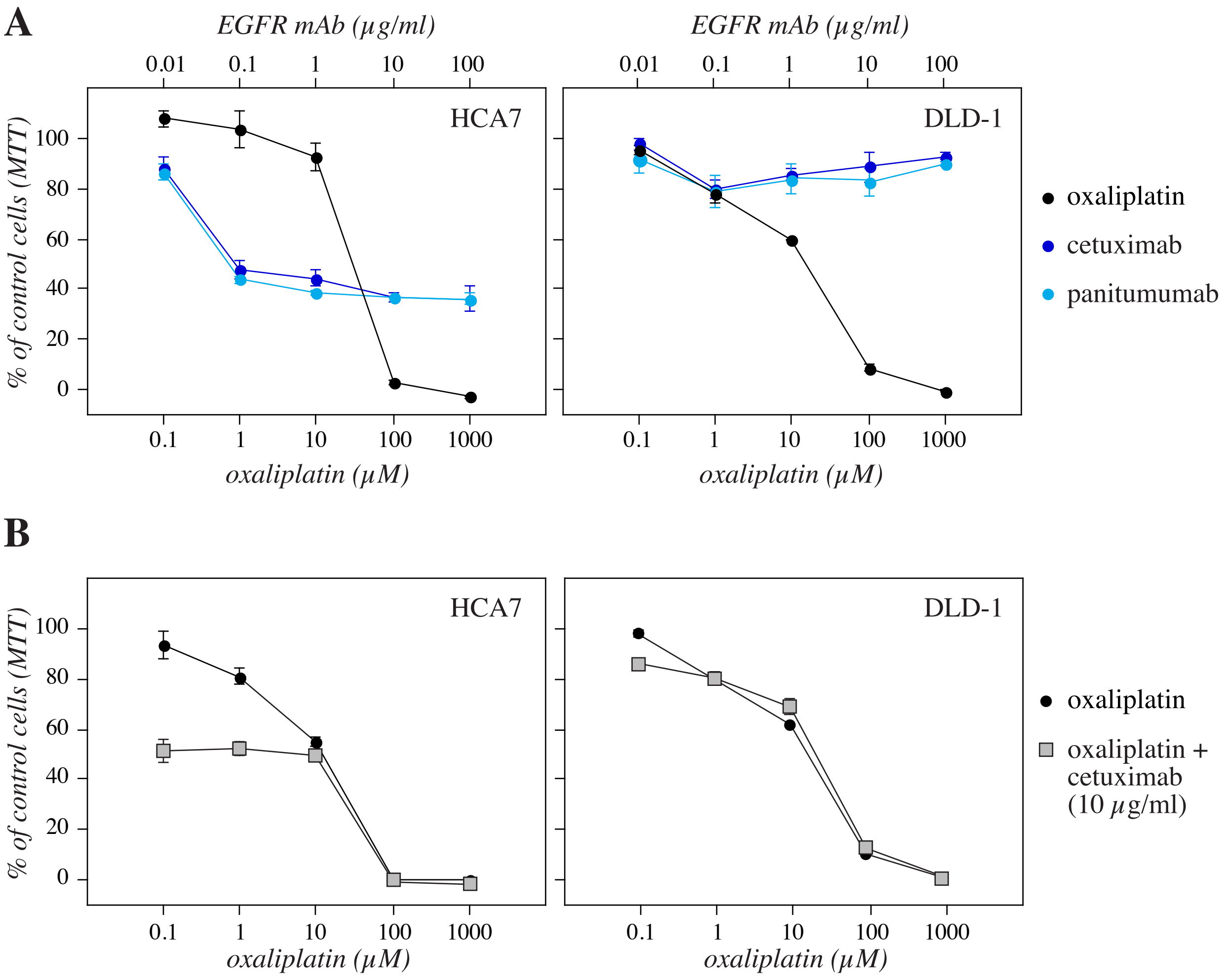

Supplementary Figure 1. Administration of EGFR mAbs and oxaliplatin alone and in simultaneous combination. The sensitivity of CRC cell lines to a 72 hour treatment with the indicated concentrations of EGFR mAbs, oxaliplatin, or their combination was tested by MTT cell viability assays. **A**) The EGFR mAb-sensitive HCA7 (left panel) and the EGFR mAb-insensitive DLD-1 (right panel) cells were treated with the EGFR mAbs cetuximab or panitumumab, or oxaliplatin as single agents. **B**) The EGFR mAb-sensitive HCA7 (left panel) and the EGFR mAb-insensitive DLD-1 (right panel) cells were treated with a simultaneous combination of cetuximab and oxaliplatin. Data are expressed as the relative MTT signal compared to untreated control cells. Mean +/– SD is shown.

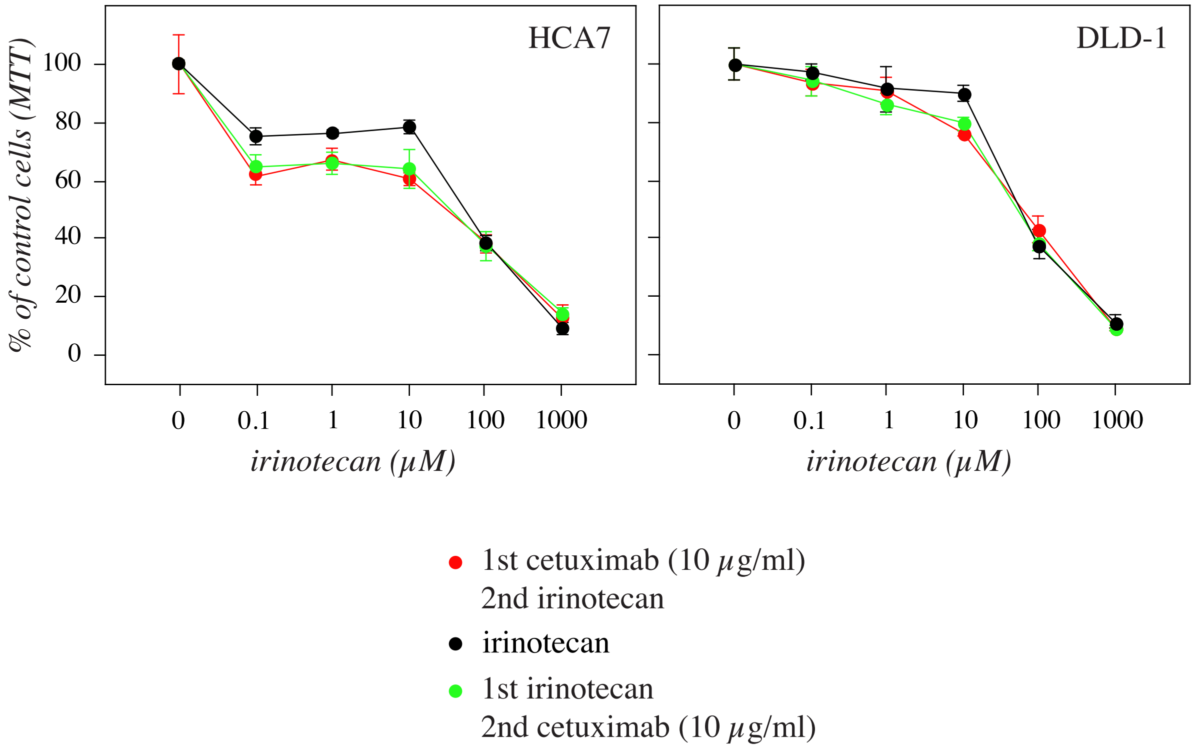

Supplementary Figure 2. Sequential administration of cetuximab and irinotecan. Sequential administration of cetuximab before or after irinotecan was compared to administration of irinotecan alone with MTT assays of CRC lines. HCA7 (**A**) and DLD-1 (**B**) cells were 1) treated for 24 hours (for day 3 of the experiment) with the indicated concentrations of irinotecan alone (black curves), 2) treated first for 48 hours (days 1 and 2) with 10 µg/ml cetuximab followed by 24 hour (day 3) treatment with the indicated concentrations of irinotecan (red curves), or 3) treated first for 24 hours (day 3) with irinotecan followed by 48 hour (days 4 and 5) treatment with 10 µg/ml cetuximab (green curves). MTT analysis was carried out after day 5 of the experiment. The mean +/– SD are shown.

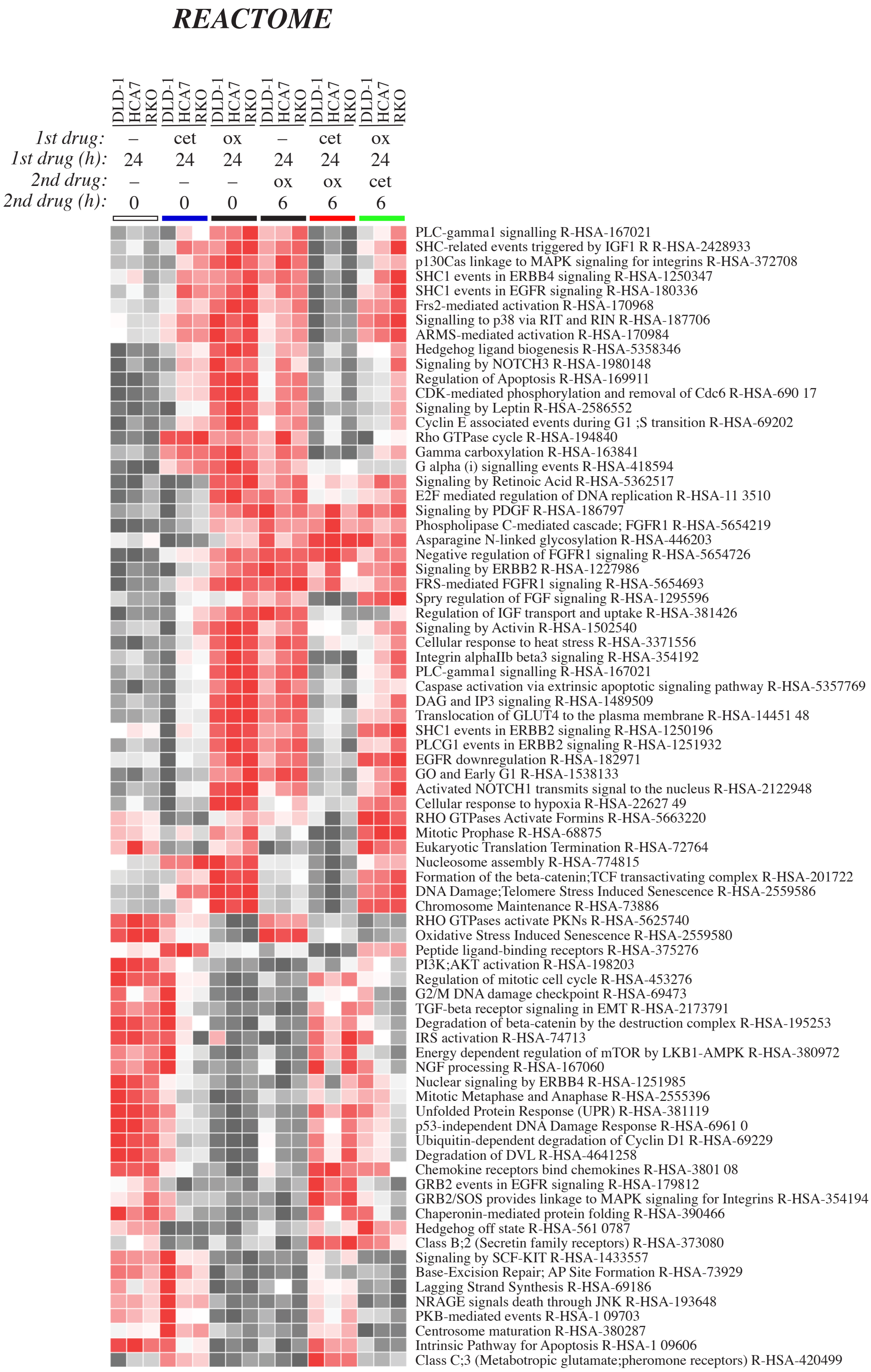

Supplementary Figure 3. RNAseq gene expression analysis by pathways annotated using Reactome database. DLD-1, HCA7, and RKO cells were subjected to different regimens containing 10 µg/ml cetuximab and/or 50 µM oxaliplatin: 1) control medium for 24 hours (white bar), 2) cetuximab for 24 hours (blue bar), 3) oxaliplatin for 24 hours (left black bar), 4) control medium for 24 hours followed by oxaliplatin for 6 hours (right black bar), 5) cetuximab for 24 hours followed by oxaliplatin for 6 hours (red bar), or 6) oxaliplatin for 24 hours followed by cetuximab for 6 hours (green bar). Gene expression was analyzed using RNAseq. Data were sectioned into pathway matrices according to the annotations of the Reactome database and converted into a heatmap.

WIKIPATHWAYS

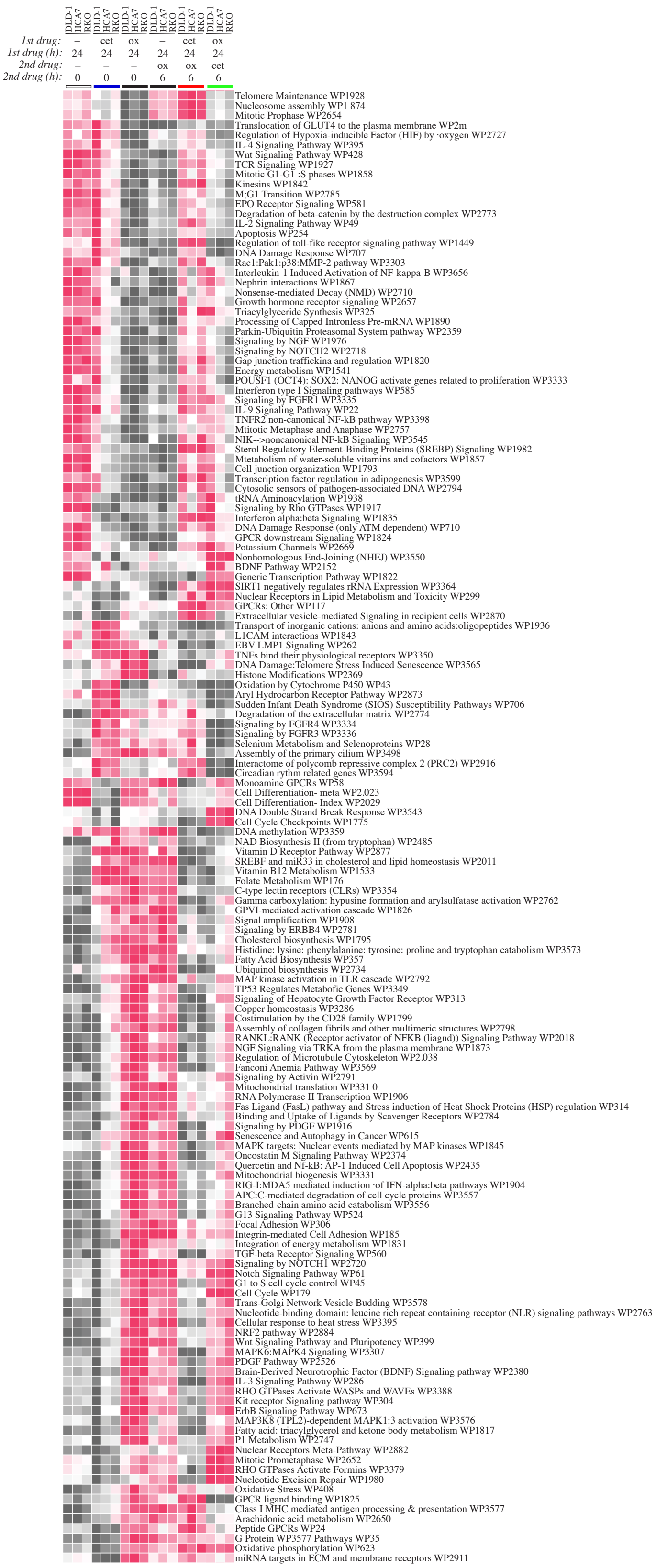

Supplementary Figure 4. RNAseq gene expression analysis by pathways annotated using Wikipathways database. DLD-1, HCA7, and RKO cells were subjected to different regimens containing 10 μg/ml cetuximab and/or 50 μM oxaliplatin: 1) control medium for 24 hours (white bar), 2) cetuximab for 24 hours (blue bar), 3) oxaliplatin for 24 hours (left black bar), 4) control medium for 24 hours followed by oxaliplatin for 6 hours (right black bar), 5) cetuximab for 24 hours followed by oxaliplatin for 6 hours (red bar), or 6) oxaliplatin for 24 hours followed by cetuximab for 6 hours (green bar). Gene expression was analyzed using RNAseq. Data were sectioned into pathway matrices according to the annotations of the Wikipathways database and converted into a heatmap.

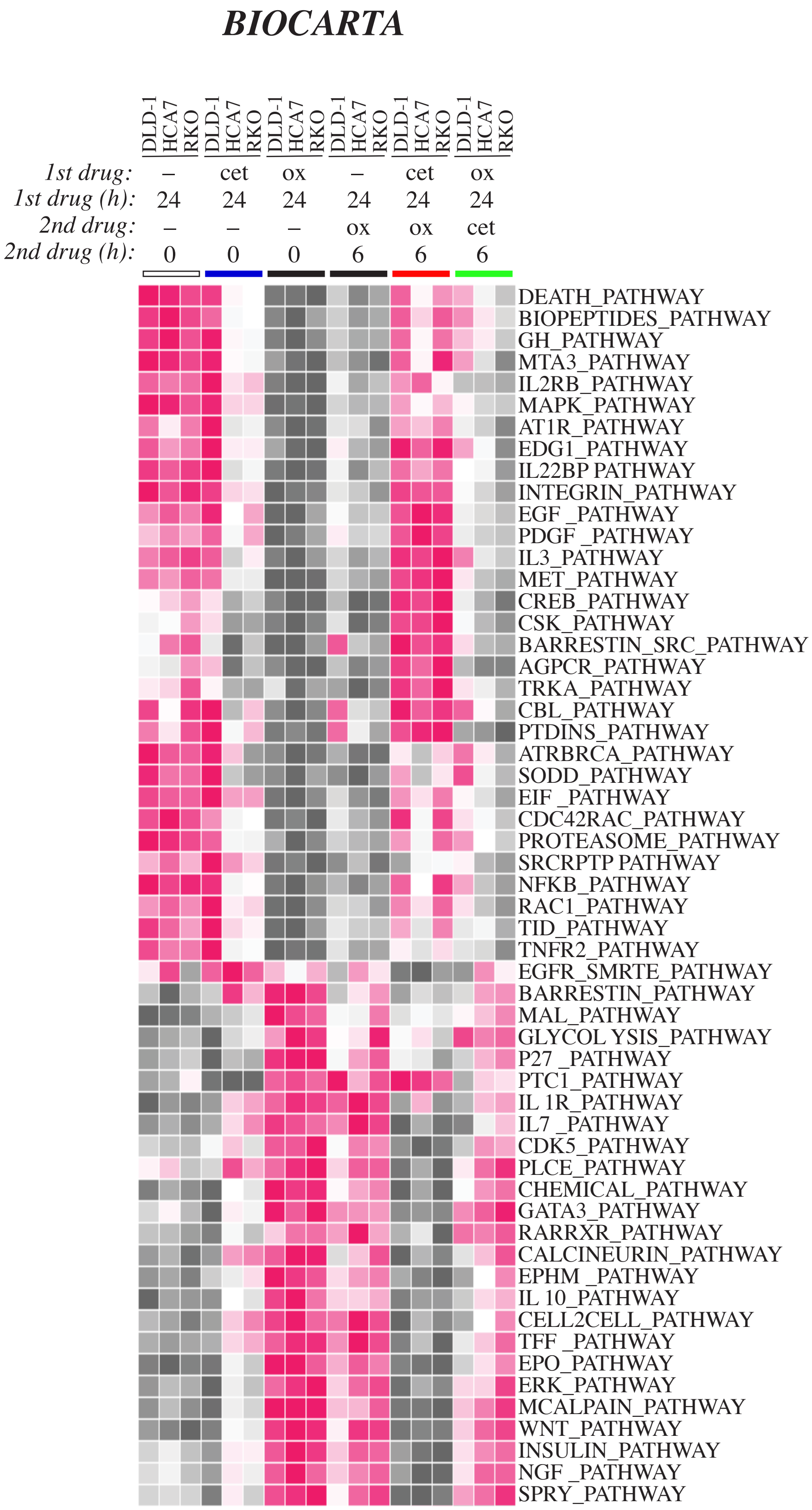

Supplementary Figure 5. RNAseq gene expression analysis by pathways annotated using Biocarta database. DLD-1, HCA7, and RKO cells were subjected to different regimens containing 10 μg/ml cetuximab and/or 50 μM oxaliplatin: 1) control medium for 24 hours (white bar), 2) cetuximab for 24 hours (blue bar), 3) oxaliplatin for 24 hours (left black bar), 4) control medium for 24 hours followed by oxaliplatin for 6 hours (right black bar), 5) cetuximab for 24 hours followed by oxaliplatin for 6 hours (red bar), or 6) oxaliplatin for 24 hours followed by cetuximab for 6 hours (green bar). Gene expression was analyzed using RNAseq. Data were sectioned into pathway matrices according to the annotations of the Biocarta database and converted into a heatmap.

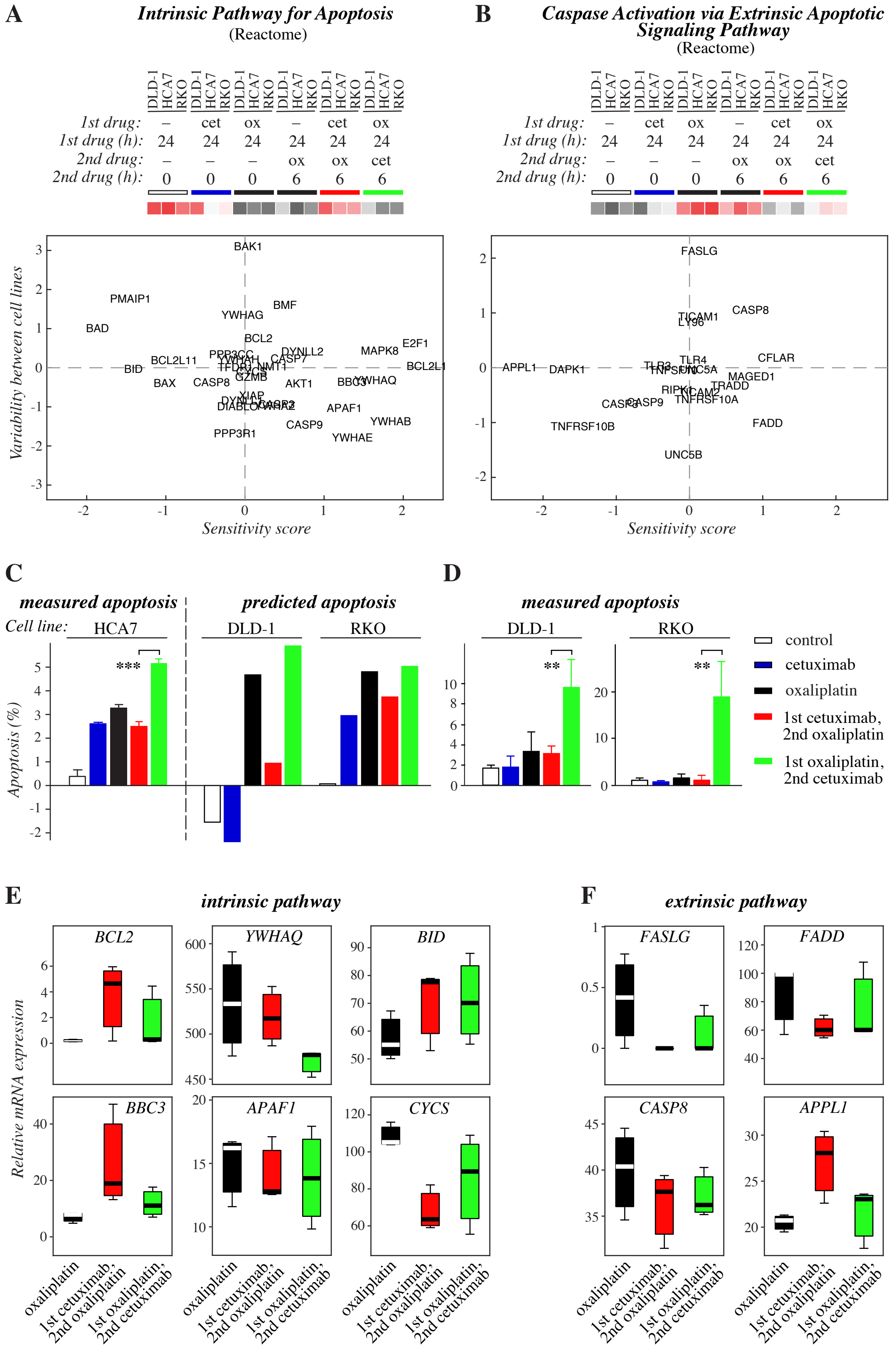

Supplementary Figure 6. Gene expression changes in RNAseq analysis predict apoptotic response for "cetuximab after oxaliplatin". Apoptosis measured by annexin V analysis of the HCA7 cells (Fig. 2B) and the expression of apoptosis-related genes in two Reactome-annotated pathways (**A, B**) were used to create a model to predict apoptosis for the two other cell lines, DLD-1 and RKO, included in the RNAseq analysis (**C**). **D**, Apoptosis measured by annexin V analysis of DLD-1 and RKO cells. **, $P < 0.01$. **E** and **F**, box plot presentations of the 10 genes that best predicted the measured apoptosis of HCA7 cells and were subsequently used to model the apoptosis of DLD-1 and RKO cells as shown in panel **C**. The box plots indicate the median (thick horizontal line), the second and third quartiles (the box), and the range (whiskers) of the data.

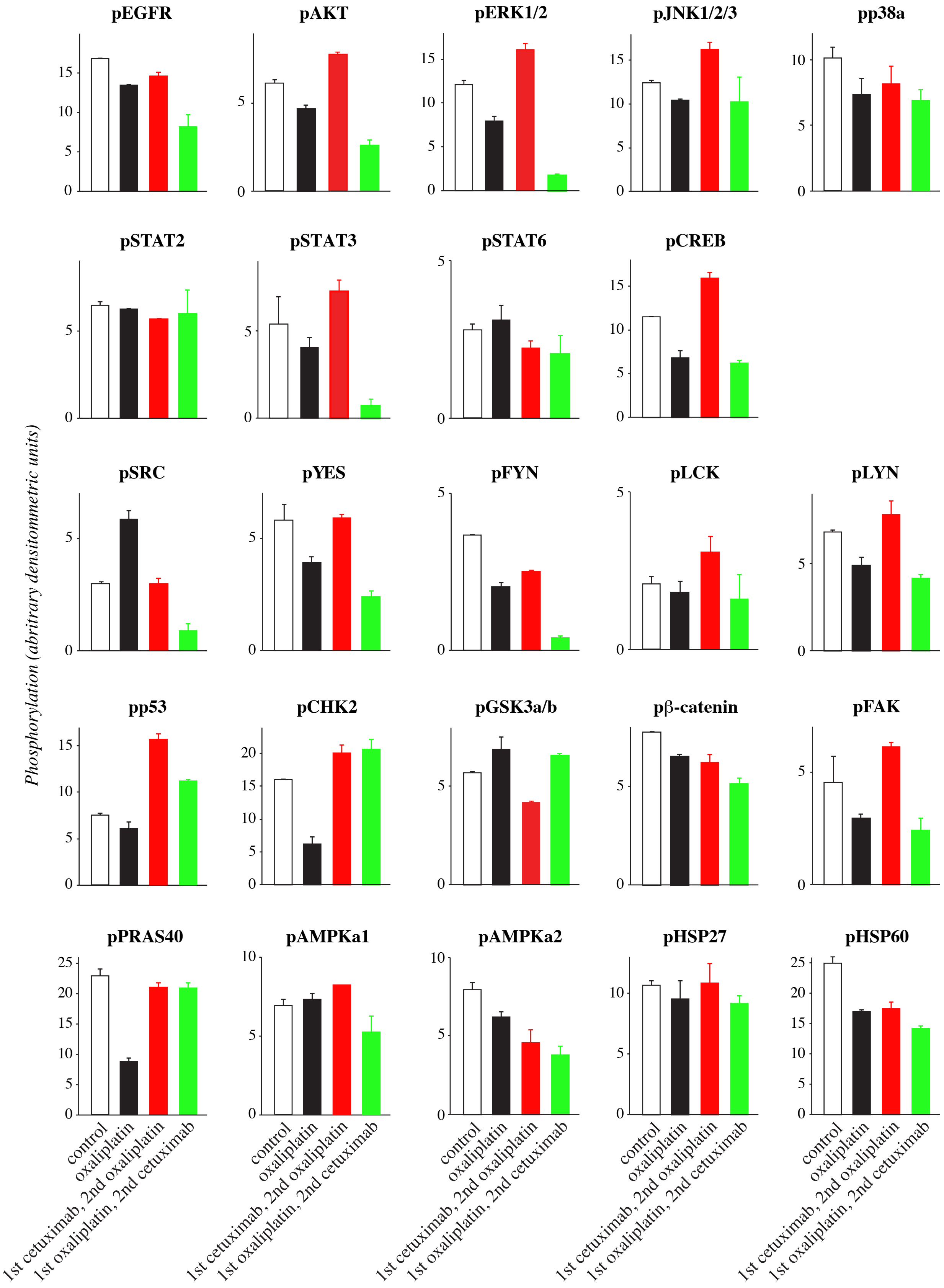

Supplementary Figure 7. Phosphoarray analysis of sequential treatments. HCA7 cells were subjected to different regimens containing 10 µg/ml cetuximab and/or 50 µM oxaliplatin: 1) control medium for 24 hours (white), 2) control medium for 24 hours followed by oxaliplatin for 1 hour (black), 3) cetuximab for 24 hours followed by oxaliplatin for 1 hour (red), or 4) oxaliplatin for 24 hours followed by cetuximab for 1 hour (green). Phosphorylation profiles were analyzed using Proteome Profiler Human Phospho-Kinase Array Kit and quantified by densitometry. Mean +/– range is shown.

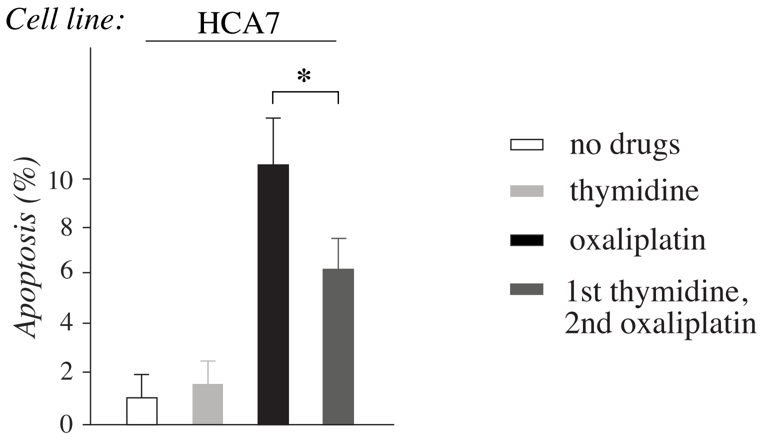

Supplementary Figure 8. Effect of G1 arrest by thymidine on oxaliplatin-induced apoptosis. HCA7 cells were arrested in G1 by 8 hour treatment with or without 2 mM thymidine followed by 18 hour treatment with or without 50 μM oxaliplatin. Apoptosis was measured by annexin V analysis. Mean +/– SD is shown for three independent experiments. *, $P < 0.05$.

Blots in Fig. 4B

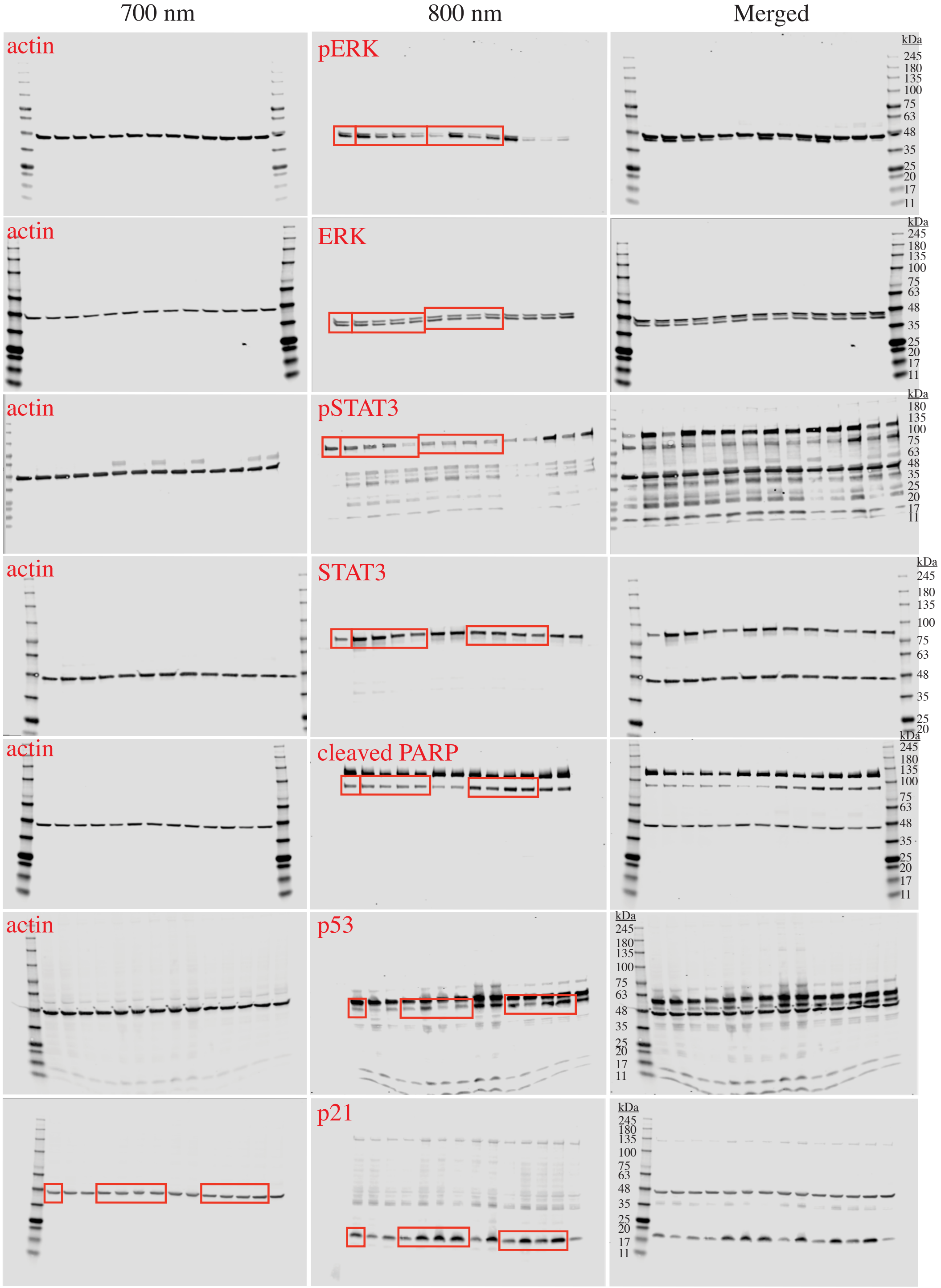

Blots in Fig. 4D

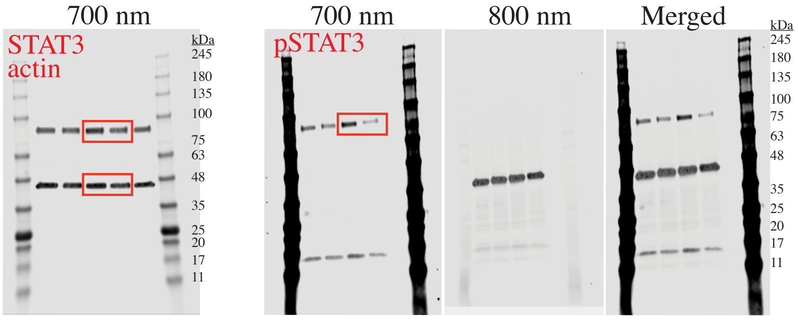

Supplementary Figure 9. Full length blots of figure 4B and 4D.
